# Effects of *N*-Acetyl-Cysteine Supplementation through Drinking Water on the Glutathione Redox Status during the Weaning Transition of Piglets

**DOI:** 10.3390/antiox8010024

**Published:** 2019-01-16

**Authors:** Jeroen Degroote, Noémie Van Noten, Wei Wang, Stefaan De Smet, Joris Michiels

**Affiliations:** Laboratory of Animal Nutrition and Animal Product Quality (LANUPRO), Department of Animal Sciences and Aquatic Ecology, Ghent University, Coupure Links 653, 9000 Ghent, Belgium; noemie.vannoten@ugent.be (N.V.N.); vei.wang@hotmail.com (W.W.); stefaan.desmet@ugent.be (S.D.S.); joris.michiels@ugent.be (J.M.)

**Keywords:** weaned pigs, glutathione, redox status, *N*-acetyl-cysteine, small intestine

## Abstract

This study investigated the effect of *N*-acetyl-cysteine (NAC) supplementation through drinking water on animal performance and the glutathione (GSH) redox system in weaned piglets, particularly in relation to the immediate post-weaning feed intake. To this end, 168 piglets were weaned and either fed *ad libitum* or fasted the first two days, and either or not administered 200 mg/L NAC via the drinking water until d14 post-weaning. Next to animal performance until day 42 (d42), the GSH redox system was measured in erythrocytes, small intestinal mucosa, liver, lung, and kidney tissue at d0, d2, and d14 post-weaning. Animal performance and GSH levels were not affected by NAC, nor by fasting. Irrespective of treatment, a significant drop in GSH at d2 post-weaning was found as compared to d0, in particular in liver (−69%), distal jejunal mucosa (−72%), and lung tissue (−80%). Post-weaning changes of the GSH redox status were strongly tissue-dependent. To conclude, this research indicates that GSH redox homeostasis was largely affected in multiple organs during the weaning transition. NAC supplementation did not increase GSH levels in any tissue, not even in fasted animals, questioning the fact if cysteine is the first or only limiting factor determining the rate of GSH synthesis in the early post-weaning phase.

## 1. Introduction

Increasing evidence implicates that weaning triggers the occurrence of oxidative stress and redox imbalance in piglets [1,2]. Next to oxidative damage to critical cell components, disturbance of the redox homeostasis is considered to influence redox signaling [3,4]. For example, in relation to gut health, redox-sensitive signaling pathways are involved in the regulation of the mucosal function [5,6]. Newly weaned piglets, thus, might have a higher need for antioxidant protection during this phase. Here, supporting the synthesis of glutathione (GSH), the most abundant endogenous antioxidant, could be a valuable approach [7]. Cells tightly regulate the synthesis of GSH, which involves two ATP-requiring enzymatic steps. The first and most important step is the γ-linkage of glutamate with cysteine, catalyzed by glutamate cysteine ligase (GCL). This step is considered rate limiting, since cysteine is generally the limiting precursor [8], and since GCL activity is controlled at multiple levels [9]. In redox reactions, GSH serves as an electron donor, resulting in the generation of its oxidized counterpart glutathione disulphide (GSSG). The redox status of the GSH/GSSG redox couple can be expressed via its half-cell redox potential (GSH/GSSG E_h_). As a result, an increased GSH/GSSG E_h_ can be interpreted as a more oxidized cell environment [3]. While almost all mammalian cells can synthesize GSH, its production might not be sufficient to maintain cellular function during certain stressful events [10]. Connected to this, sulfur amino acid requirements of piglets are considered to increase in inflammatory conditions [11,12]. Therefore, supplementing the diet with cysteine could have beneficial effects in weaned piglets. Here, the *N*-acetyl derivative of this amino acid, *N*-acetyl-cysteine (NAC) could be preferred since the acetyl-substituted amino group prevents cysteine from being oxidized to the insoluble cystine in the gastro-intestinal tract [13]. Absorption and de-acetylation of NAC in the small intestine is almost complete [14], making cysteine readily available for GSH synthesis. Due to these reasons, NAC has been suggested as a nutritional intervention to remedy weaning-related health issues [15,16,17,18,19,20]. For example, Xu et al., (2014) found decreased intestinal levels of malondialdehyde, H_2_O_2_, and NO when administering NAC in a 500 mg/kg diet, and further correlated these changes with the proliferation of selected microbial groups in the gut [15]. Hou et al. (2011) showed that dietary NAC supplementation (500 mg/kg diet) could partially ameliorate lipopolysaccharide (LPS) induced damage to the small intestinal mucosa of weaned piglets [18]. These beneficial effects are not believed to originate from the direct radical scavenging activity of NAC [21], but are considered to be mediated by the GSH redox system [22], as was also indicated by Hou et al. (2013) in a follow-up study [17]. However, none of the previously mentioned studies monitored or reported this GSH response. Furthermore, and it remains unclear if NAC can increase tissue GSH levels in piglets as early as two days post-weaning. This early weaning period is particularly characterized by inadequate feed intake [23,24] and, thus, a deficit in a large number of essential nutrients. In this phase, the effect of NAC on the GSH redox status remains unknown, especially in relation to the inadequate nutritional status of the weaned piglet. Hence, the current study aimed to evaluate if NAC at similar doses as previous research [15,16,17,18,19,20] could increase GSH levels in tissues in weaned piglets. Further, we aimed to evaluate if this effect is dependent on the nutritional status of the animal during the early weaning transition.

## 2. Materials and Methods 

### 2.1. Experimental Animals and Feeds

The experiment was carried out according to the guidelines of the Ethical Committee of the Faculty of Veterinary Sciences, Ghent University (Belgium) for the humane care and use of animals in research (Ethical approval number: 2015/11). For this experiment 168 piglets (Topigs hybrid × Piétrain) were selected at weaning from 21 litters at one farm. Barrows and gilts (4.5–9.5 kg) were weaned at 26 days of age, and were individually examined for clinical signs of disease, such as lethargy, lameness, diarrhea, and respiratory diseases. Sick animals were excluded from the experiment. Selected piglets were transported to the trial facilities and allocated to the pens and treatments according to procedures described below. Six piglets were housed per pen (2.10 m^2^/pen) with full slatted floors, conventional ventilation scheme, starting ambient temperature at 30 °C and a 24 L schedule until d5 post-weaning. Hereafter, ambient temperature was linearly adjusted to 26 °C with 18L:6D light schedule. All piglets were fed the same commercial weaner diet and starter diet (AVEVE Veevoeding, Merksem, Belgium). Diets were wheat- and soy-based meals, containing no in-feed antibiotics or pharmacological doses of zinc oxide. The weaner and starter diets were respectively offered from d0–14 and d14–42 post-weaning. A more detailed description can be found in Table 1.

### 2.2. Experimental Design and Measurements

Two study factors were included in a 2 × 2 full factorial design. Piglets were either or not supplemented with NAC via the drinking water, and either or not fasted (FA) during the first 48 h of the experiment. Fasted animals still had access to drinking water, but not to feed during that period. The four resulting experimental treatments were as follows: *ad libitum* access to feed and drinking water without NAC (FA−NAC−); *ad libitum* access to feed and drinking water supplemented with 200 mg/L NAC during d0–14 post-weaning (FA−NAC+); no access to feed during d0–2 post-weaning and *ad libitum* access to drinking water without NAC (FA+NAC−); no access to feed during d0–2 post-weaning and *ad libitum* access to drinking water supplemented with 200 mg/L NAC during d0–14 post-weaning (FA+NAC+). Treatments were replicated in seven pens with six piglets per pen. First, piglets were allocated to the different pens in order to stratify for body weight and gender. Then, treatments were allocated to the pens according to a randomized block design. Piglets were weighed individually at d0, d2, d14, and d42 post-weaning. Concurrently, feed intake was registered at the pen level by weighing feeders and residual feed. Water intake was measured daily on pen level, as described below. Average daily gain (ADG), average daily feed intake (ADFI), average daily water intake (ADWI), feed conversion ratio (FCR) and water to feed ratio (WFR) were calculated for following intervals; d0–2, d2–d14, d14–42, and d0–42 post-weaning. 

Animals were inspected daily by the same trained individual for general health and clinical observations (fecal consistency and diarrhea incidence). A fecal consistency score of each pen was assessed visually at 10 am throughout the 14d post-weaning period, according to an ordinal scoring system consisting of three categories: 1 = hard or slightly moist feces, clearly formed, normal; 2 = moist or soft feces, but still with a definite form, sticky; and 3 = watery or liquid feces, unformed, diarrhea. Color of the feces was not included as scoring criteria. The frequency of diarrhea incidence was simultaneously assessed by counting pigs with a fecal consistency score of 3, including clear signs of diarrhea: filthy, wet backside and tail, dehydrated, loss of condition, and irritation of the skin around the anus. Antibiotic treatments were registered and was limited to individual intramuscular injections. 

### 2.3. Water Supplementation

NAC (CAS 616-91-1) was obtained from Zambon (Zambon N.V., Jette, Belgium) as a ≥99% grade powder. Supplementation of NAC was performed by an automated water additive dispenser (HQ-MultiMix, Waterschoot Hq-line BVBA, Meerhout, Belgium). Here, a working solution of 20,000 mg/L NAC was supplemented at a rate of 1% of the water consumption, resulting in a final concentration of 200 mg/L drinking water. The working solution was continuously agitated by a magnetic stirrer (200 RPM, no heating) and was prepared fresh on a daily basis. Water pressure in the installations was controlled at 1 bar and water was available via one bowl drinker with a standard nipple (Drink-O-Mat mini, MS Schippers, Arendonk, Belgium) per pen. Two identical water lines, constructed out of 20 mm diameter PVC pressure pipes and fittings, were available to either provide standard tap water or NAC supplemented water to each individual pen. The single drinker of each pen was equipped with a high performance water gauge (HQ-liter check, Waterschoot Hq-line BVBA, Meerhout, Belgium), and a non-return valve. The gauges were classified as accuracy class C (ISO 4046), had a nominal water flow (Qn) of 1 m^3^/h and a start flow of 0.1 L/min. Water consumption was recorded daily at 10 am by registering the gauge display, which had a minimum readout of 0.1 L.

### 2.4. Sample Collection 

Four piglets from the same batch were not part of the experiment, but were sampled at d0, immediately upon arrival to the weaning facilities. At d2 and d14 post-weaning, four piglets per treatment, having the median body weight within the pen at that time, were selected for euthanasia and sampling. In one pen per treatment, one animal was selected both at d2 and d14 post-weaning. In the six remaining pens per treatment, only one animal was selected for sampling either at d2 or d14 post-weaning. No fasting period was included for *ad libitum* fed piglets. Animals were anaesthetized by electrical stunning and during exsanguination, blood was collected in heparinised tubes containing 1 mM bathophenanthroline disulfonic acid. Unclotted blood was immediately centrifuged (3000× *g,* 10 min) and then the supernatant was removed. Erythrocytes were lysed with metaphosphoric acid and intense vortexing. After centrifugation (3000× *g*, 10 min), a portion of the resulting acid extract was supplemented with γ-glutamyl-glutamate, snap frozen and stored at −80 °C. Meanwhile, the gastro-intestinal tract was removed, the small intestine was isolated, and its length was determined. A 20 cm section was obtained at both 5% (duodenum) and 75% (distal jejunum) of the total small intestinal length. Sections were rinsed with 0.9% saline and further processed on an ice cold surface. The tissues were slit longitudinally, and scraped with glass slides for mucosa collection. A 1 g mucosa subsample was homogenized in 10 mL ice cold 1.16 M perchloric acid with a Braun Potter S homogenizer (1000 rpm, 30 sec). After centrifugation (10,000× *g*, 15 min), an aliquot of the supernatant was transferred, supplemented with γ-glutamyl-glutamate, snap frozen in liquid nitrogen and stored at −80 °C pending quantification of GSH and GSSG.

Next, the liver was isolated and a subsample was taken central in the right lateral lobe. Following, the right kidney was removed from the abdominal cavity. After removing the capsule, a 0.5 cm slice was made in the coronal plane with a surgical blade. The renal cortex was identified as the dark red outmost layer of the kidney, and was separated from the slightly paler medulla by dissection. Only renal cortical tissue was subsequently used for analysis. Finally, the lungs were removed from the thoracic cavity and a tissue segment was excised at the diaphragmatic surface in the middle of the right caudal lung lobe. Liver, kidney and lung tissue were rinsed with ice cold saline, blotted dry, and minced with a surgical blade. For each organ, a 2 g tissue subsample was turned into an acidified homogenate, according to the procedure described for mucosa.

### 2.5. Laboratory Analysis

The methodology of Yoshida et al. (1996) was followed to determine GSH and GSSG in the different tissues [25]. In brief, iodoacetic acid was used as thiol quenching agent, and the acid solution was brought to pH 8–9 by the addition of a potassium hydroxide-potassium bicarbonate buffer. Overnight incubation with 1-fluoro-2,4-dinitrobenzene resulted in stable thiol derivates that were separated on an aminopropyl column by reverse-phase HPLC. Chromatographic runs were performed with water/methanol (1:4 *v*/*v*) and acetic acid (0.5 M)/methanol (1:1.78 *v*/*v*) as mobile phases, and with UV absorption measurement at 365 nm. The GSH and GSSG concentrations were determined relative to γ-glutamyl-glutamate as internal standard and to GSH and GSSG external standard solutions. Results were expressed on wet tissue weight basis. The GSH/GSSG E_h_ values (in mV) were calculated by using the appropriate forms of the Nernst equation, at pH 7.4 and temperature of 37 °C, whereby concentrations of GSH and GSSG were expressed in molarity [3]:

GSH/GSSG E_h_ = −264 − 61.5/2 × log_10_ (GSH^2^/GSSG)

### 2.6. Statistics

The experimental unit for animal performance was the pen. The individual animal was considered the experimental unit for analytical variables. All tests were performed in IBM SPSS Statistics version 24.0 (SPSS Inc., Chicago, IL, USA). Data were checked for violations on the normality and homoscedasticity assumptions, before applying an analysis of variance (ANOVA) according to the general linear model module. Factors FA and NAC were included as fixed effects, together with the effect of days post-weaning (DAY) if appropriate. Data are presented as least squares means with the standard error of the mean (SEM) in tables, or with the standard deviation (SD) in figures. Differences were considered significant at *p* ≤ 0.05, and statistical tendencies were assumed when 0.10 > *p* > 0.05. Tukey’s multiple comparison test was applied to compare means of more than two treatment groups. For significant three-way interactions, the differences were inspected by post-hoc analysis along all different treatment combinations.

Principal component analysis (PCA) was used as a dimension reduction technique, to evaluate if treatments groups had altered overall oxidative/redox status. First, the significance (at *p* ≤ 0.05) of linear correlations between all variables was checked by means of bivariate Pearson correlation coefficients. Following this, data from 12 variables at d0, d2 and d14 post-weaning were included in the PCA, being the GSH and GSSG concentrations in erythrocytes, duodenal mucosa, distal jejunal mucosa, liver, lung, and the renal cortex. The GSH/GSSG E_h_ was not included since this variable is computed from the GSH and GSSG concentrations, and might, therefore, result in multicollinearity. The subject-to-variable ratio was 3 to 1. First, data were standardized, i.e., for each observation the value was subtracted with the grand mean and divided by the standard deviation. An initial PCA gave rise to three principal components with an eigenvalue higher than 1. The first two principal components, with initial eigenvalues of 5.6 and 1.9, were retained for a final PCA. Here, the Kaiser-Meyer-Olkin statistic of sampling adequacy was 0.687. Bartlett’s test of sphericity (*p* < 0.001) indicated multivariate normal distribution with zero covariance. Varimax rotation with Kaiser normalization ensured maximal sum of the variances of the squared loadings matrix. Variables with |r| < 0.5 are not shown in the result section, but were not suppressed in the PCA. Piglets received component scores for the two principal components by using the least squares regression approach. Similar ANOVA principals as described above were used to evaluate treatment effects on scorings of those two final components.

## 3. Results

### 3.1. Animal Performance

In general, growth performance was good for conditions set, e.g. final BW easily reached 22 kg at 42d post-weaning. No animals had to be removed from the study, antibiotic treatments were limited to a few individual intramuscular injections, and no mortality occurred during the experiment. Incidence of diarrhea during the first 14 d post-weaning was low, as on a daily basis only 1.6% of the animals showed clear indications of diarrhea after thorough individual inspection. A moderate surge in diarrhea incidence was observed during d4–8 post-weaning, but this seemed not to be treatment related (*p* > 0.05) (data not shown). The average fecal consistency score however tended to be higher in NAC-supplemented piglets (*p* = 0.098), where an increase from 1.19 (NAC−) to 1.33 (NAC+) was observed on a scale from 1 to 3 (data not shown). More elaboration on animal performance and treatment differences can be found in Table 2. Final BW and overall ADG, ADFI, ADWI, FCR, and WFR were not affected by the different treatment factors (FA and NAC), nor by an interaction of those factors (FA × NAC). This also holds true for the latest phase of the experiment (d14–42 post-weaning).

Still, during the first phase of the experiment (d0–2 post-weaning) some minor treatment differences were observed. For instance, BW was reduced with 4.8% in FA+ piglets at d2 with respect to d0, compared to 1.9% in FA− piglets (*p* < 0.001). This difference most obviously resulted from the experimental setup, were FA+ piglets were fasted during the first 48h post-weaning. It must be noted that feed intake was very low in FA− piglets, and only reached an ADFI of 53 g/d. This value is far lower than the difference in ADG between FA+ and FA− piglets, which amounted to app. 100 g/d. Additionally, fasting did not affect the water intake during the fasting period (*p* = 0.899). Likewise, NAC supplementation did not significantly influence ADWI during d0–2 post-weaning, nor was there a significant treatment interaction (FA × NAC; *p* = 0.954). Further, even though supplementation of the drinking water with NAC at 200 mg/L was maintained until d14 of the trial, NAC addition did not affect water intake (*p* = 0.920) during this phase. Finally, regarding the feed efficiency (FCR) and the water to feed ratio (WFR), no significant treatment effects or treatment interactions were observed during d0–2 and d2–14 post-weaning.

### 3.2. Responses of the Glutathione Redox System in Different Tissues

**Figure 1:**
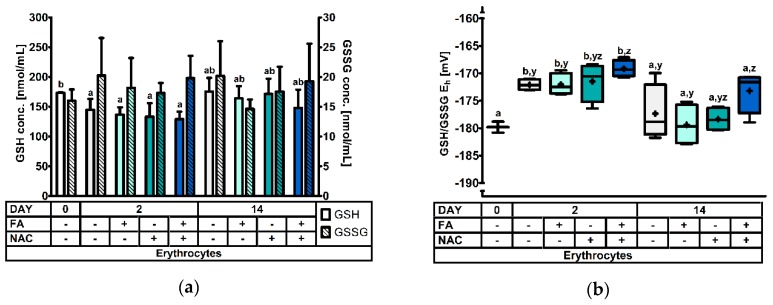
The glutathione redox system in erythrocytes of piglets at d0, d2, and d14 post-weaning (DAY), either *ad libitum* fed (FA−) or fasted (FA+) the first 2d post-weaning, and either provided standard drinking water (NAC−) or water supplemented with 200 mg/L *N*-acetyl cysteine (NAC+) during the first 14d post-weaning. Results are presented as least squares means with SD. Significance levels of main effects and interaction terms are presented in Table 3: (**a**) Erythrocyte glutathione (GSH) and glutathione disulphide (GSSG) concentrations. ^a,b^ Represent the effect of DAY across other factors (*p* ≤ 0.05); (**b**) Erythrocyte glutathione redox status (GSH/GSSG E_h_). ^a,b^ Represent the effect of DAY across other factors (*p* ≤ 0.05). ^y,z^ Represent the effect of FA × NAC across DAYS (*p* ≤ 0.05).

**Figure 2:**
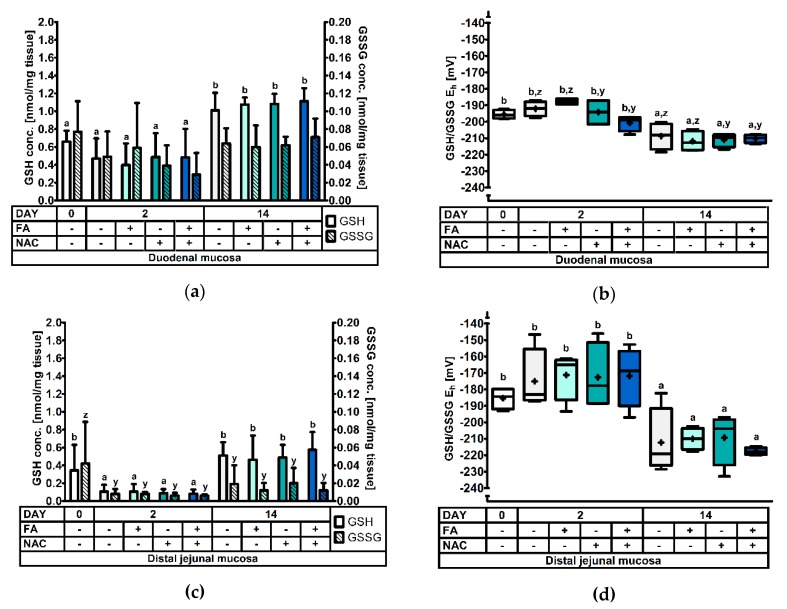
The glutathione redox system in the small intestinal mucosa of piglets at 0d, 2d, and 14d post-weaning (DAY), either *ad libitum* fed (FA−) or fasted (FA+) the first 2d post-weaning, and either provided standard drinking water (NAC−) or water supplemented with 200 mg/L *N*-acetyl cysteine (NAC+) during the first 14d post-weaning. Results are presented as least squares means with SD. Significance levels of main effects and interaction terms are presented in Table 3: (**a,c**) Glutathione (GSH) and glutathione disulphide (GSSG) concentrations in the duodenal mucosa (**a**) or the distal jejunal mucosa (**c**). ^a,b^ And ^y,z^ respectively represent the effect of DAY on GSH and GSSG concentrations across other factors (*p* ≤ 0.05); (**b,d**) Glutathione redox status (GSH/GSSG E_h_) in the duodenal mucosa (**b**) or the distal jejunal mucosa (**d**). ^a,b^ and ^y,z^ respectively represent the effect of DAY and NAC across other factors (*p* ≤ 0.05).

**Figure 3:**
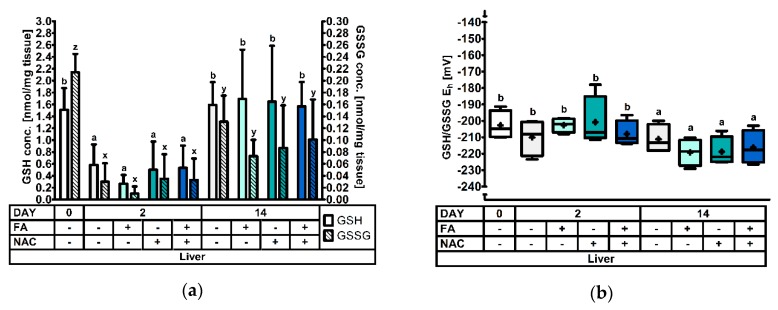
The glutathione redox system in liver tissue of piglets at 0d, 2d, and 14d post-weaning (DAY), either *ad libitum* fed (FA−) or fasted (FA+) the first 2d post-weaning, and either provided standard drinking water (NAC−) or water supplemented with 200 mg/L *N*-acetyl cysteine (NAC+) during the first 14d post-weaning. Results are presented as least squares means with SD. Significance levels of main effects and interaction terms are presented in Table 3: (**a**) Hepatic glutathione (GSH) and glutathione disulphide (GSSG) concentrations. ^a,b^ and ^x,y,z^ respectively represent the effect of DAY on GSH or GSSG concentrations across other factors (*p* ≤ 0.05); (**b**) Hepatic glutathione redox status (GSH/GSSG E_h_). ^a,b^ represent the effect of DAY across other factors (*p* ≤ 0.05).

**Figure 4:**
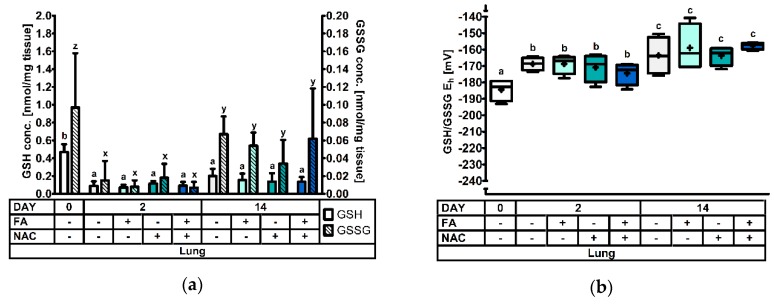
The glutathione redox system in lung tissue of piglets at 0d, 2d, and 14d post-weaning (DAY), either *ad libitum* fed (FA−) or fasted (FA+) the first 2d post-weaning, and either provided standard drinking water (NAC−) or water supplemented with 200 mg/L *N*-acetyl cysteine (NAC+) during the first 14d post-weaning. Results are presented as least squares means with SD. Significance levels of main effects and interaction terms are presented in Table 3: (**a**) Pulmonary glutathione (GSH) and glutathione disulphide (GSSG) concentrations. ^a,b^ and ^x,y,z^ respectively represent the effect of DAY on GSH or GSSG concentrations across other factors (*p* ≤ 0.05); (**b**) Pulmonary glutathione redox status (GSH/GSSG E_h_). ^a,b,c^ represent the effect of DAY across other factors (*p* ≤ 0.05).

**Figure 5:**
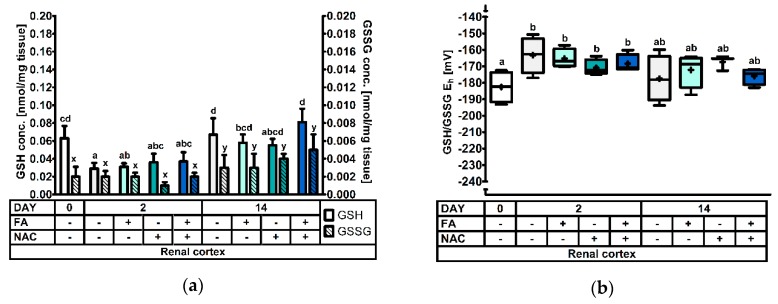
The glutathione redox system in kidney tissue of piglets at 0d, 2d and 14d post-weaning (DAY), either *ad libitum* fed (FA−) or fasted (FA+) the first 2d post-weaning, and either provided standard drinking water (NAC−) or water supplemented with 200 mg/L *N*-acetyl cysteine (NAC+) during the first 14d post-weaning. Results are presented as least squares means with SD. Significance levels of main effects and interaction terms are presented in Table 3: (**a**) Renal cortical glutathione (GSH) and glutathione disulphide (GSSG) concentrations. ^a,b,c,d^ and ^x,y^ respectively represent differences in GSH or GSSG concentrations between treatments (*p* ≤ 0.05); (**b**) Renal cortical glutathione redox status (GSH/GSSG E_h_). ^a,b^ represent the effect of DAY across other factors (*p* ≤ 0.05).

At d0, d2, and d14 post-weaning four animals per treatment were selected and sacrificed for GSH and GSSG analysis in different tissues. Significant treatment effects and interactions are indicated in Table 3. Results for the GSH and GSSG levels, and the GSH redox status are represented in Figure 1, Figure 2, Figure 3, Figure 4 and Figure 5. 

#### 3.2.1. The Erythrocyte Glutathione Redox System.

In erythrocytes, GSH concentrations were only affected by DAY (*p* = 0.002), but not by fasting, NAC-supplementation or any interaction of these factors (*p* > 0.05). The GSH concentration was highest on d0, and significantly dropped with 22% at d2 post-weaning (Figure 1a). Levels partially recovered at d14 post-weaning, but were not significantly different from d0 or d2 post-weaning. Next, even though GSSG remained unaffected, the GSH/GSSG E_h_ was altered by the day post-weaning (*p* < 0.001) and the interaction FA × NAC (*p* = 0.041). Here, the redox status was increased with approx. 7 mV at d2 post-weaning, compared to d0 and d14, indicating a more oxidized GSH redox status. Additionally, a higher GSH/GSSG E_h_ was also observed in FA+NAC+ piglets, compared to FA−NAC− and FA+NAC− piglets. This finding corresponds with the tendency for increased GSSG concentration in FA+NAC+ (*p* = 0.081) and a higher GSH/GSSG E_h_ NAC+ piglets (*p* = 0.060).

#### 3.2.2. The Glutathione Redox System in the Duodenal and Distal Jejunal Mucosa

Results on the GSH redox status of the duodenal mucosa are visualized in Figure 2a,b, significance levels can be found in Table 3. It was found that GSH levels almost doubled at d14 post-weaning, compared to d0 and d2 post-weaning (*p* < 0.001). Likewise as in erythrocytes, GSSG concentrations in the duodenal mucosa were not significantly affected by any of the factors, though tended (*p* = 0.077) to be higher on d0 than on d2 post-weaning. The duodenal GSH/GSSG E_h_ was altered by the factors DAY (*p* < 0.001) and NAC (*p* = 0.044), but not by FA (*p* = 0.552). The GSH redox status was 15 to 18 mV higher, respectively at d0 and d2 post-weaning, compared to d14. A significantly more reduced GSH redox status was also observed in NAC+ piglets (−204 mV) than in NAC− piglets (−199 mV). Remarkably, the interaction term DAY × NAC, and also the three-way interaction term DAY × FA × NAC demonstrated a statistical trend (0.05 < *p* < 0.10). However, post-hoc analysis did not reveal differential responses.

As for the distal jejunal mucosa (Figure 2c,d, Table 3), it was found that GSH level were highly depressed at d2, both compared to d0 (−72%) and d14 (−81%) post-weaning (*p* < 0.001). No other treatment factors or interactions reached significance. Furthermore, GSSG concentrations were significantly different between days post-weaning (*p* = 0.027), where levels were highest at d0, significantly dropped on d2 post-weaning, and remained at this level on d14 post-weaning. Similar to the duodenal GSH redox status, the distal jejunal GSH/GSSG E_h_ was higher at d0 (+27 mV) at d2 (+40 mV) post-weaning, compared with d14 (*p* < 0.001). These differences seemed more pronounced than in the duodenal mucosa.

#### 3.2.3. The Hepatic Glutathione Redox System 

Results for liver tissue are depicted in Figure 3 and Table 3. Here, somewhat similar responses were observed as in the distal jejunal mucosa. For instance, the GSH concentrations were severely decreased at d2, compared both to d0 (−69%) and d14 (−71%) post-weaning (*p* < 0.001). Additionally, GSSG levels were affected by DAY (*p* < 0.001). Levels were again highest on d0, and significantly dropped on d2 post-weaning. At d14, GSSG reached intermediate levels that were significantly different from d0 and d2 post-weaning. Finally also the GSH redox status demonstrated a similar pattern as in the distal jejunum. Values were significantly higher at d0 (+11 mV) and d2 (+14 mV), compared to d14 post-weaning. Apart from DAY, no other treatment factors altered GSH, GSSG or GSH/GSSG E_h_ (*p* > 0.05), only for GSH/GSSG E_h_ a tendency (*p* = 0.076) for the factor DAY × FA × NAC was observed. 

#### 3.2.4. The Pulmonary Glutathione Redox System

Figure 4 and Table 3 contain the results obtained in lung tissue. Similar as for the other sample types, GSH values dropped significantly (−80%) on d2 compared to d0 post-weaning. Conversely, concentrations remained at this low level on d14. GSSG concentrations showed a similar pattern from d0 to d2, with a profound decrease on d2 post-weaning. Contrasting to GSH, GSSG values increased almost to levels of d0 by d14. These changes are also reflected in the GSH redox status, where it was found that a more oxidized redox status was observed at d2 (+14 mV) and d14 (+24 mV), as compared to d0 post-weaning. Remarkably, this is an opposite pattern as was observed in most other tissues. Regarding the effect of FA and NAC, no significant differences were observed (*p* > 0.05).

#### 3.2.5. The Renal Cortical Glutathione Redox System

Figure 4 comprises the measurements in the cortex of the kidney. Significance levels can be found in Table 3. Renal cortical GSH was decreased to approx. 50% at d2, compared to both d0 and d14 post-weaning (*p* < 0.001). However, there was also an interaction effect observed (DAY × FA × NAC: *p* = 0.038). Additionally, the interaction FA × NAC tended (*p* = 0.055) to influence the outcome. A multiple comparison test demonstrated that particularly FA−NAC− and FA+NAC+ piglets at d14 had high GSH concentrations in the renal cortex. Conversely, GSH was lowest at d2 post-weaning in FA−NAC− piglets. No specific pattern could be deduced describing this interaction effect. Next, GSSG concentrations were also influenced by day post-weaning (*p* < 0.001) with the highest values at d14. However, the factor DAY × NAC tended to influence results (*p* = 0.082). Regarding the GSH redox status, values indicated a significantly more oxidized redox environment on d2, as compared to d0 post-weaning (+16 mV). The GSH/GSSH E_h_ on d14 was not significantly different from other d0 or d2 post-weaning. Factors FA and NAC did not influence GSSG or GSH/GSSG E_h_ (*p* > 0.05).

### 3.3. Principal Component Analysis

**Figure 6:**
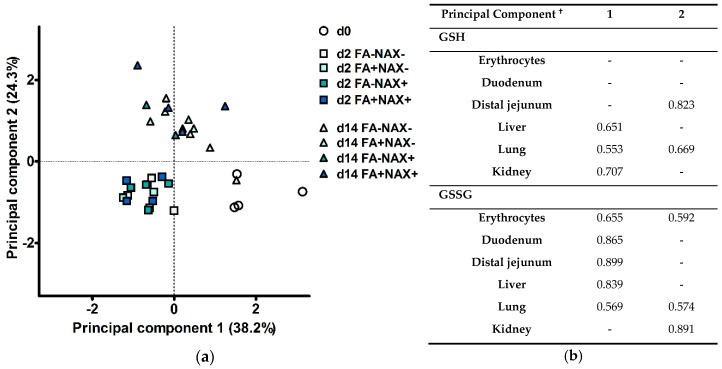
Principal component analysis of glutathione (GSH) and glutathione disulfide (GSSG) concentrations in six different tissues of piglets at 0d, 2d, and 14d post-weaning, either *ad libitum* fed (FA−) or fasted (FA+) the first 2d post-weaning, and either provided standard drinking water (NAC−) or water supplemented with 200 mg/L *N*-acetyl cysteine (NAC+) during the first 14d post-weaning: (**a**) Scores plot representing the 36 individual piglets in the multivariate space of the first two principal components. Animals can be visually clustered according to day post-weaning (d0, d2, or d14). (**b**) Loadings of the two major principal components. These loadings indicate the correlation (positive or negative) between the original variables and the principal component. Squared factor loadings indicate the percentage of variance in an original variable explained by the principal component. ^**†**^ Only correlations with |r|> 0.5 are indicated.

A PCA was executed to assess whether treatments would cluster according to the GSH redox system, and to determine relationships among the variables in the different tissues. Two principal components were extracted, explaining 38.2% and 24.3% of the total variance. Hence, 62.5% of the total variance in the 12 variables measured could be condensed into two new variables (PC). The subject score plot for PC1 vs. PC2 is shown in Figure 6a, and component loadings are presented in Figure 6b. First, a number of observations may be made related to the treatment clustering. Piglets at d0, d2, and d14 post-weaning each formed a distinct cluster. Objects did not visually cluster according to any other factor, such as FA or NAC. The clusters of d0 and d2 were mainly opposed on the principal component 1 axis. ANOVA-testing for the scorings on PC1 could indicate the effect of DAY (*p* < 0.001), and could furthermore significantly differentiate d0, d2 and d14 post-weaning from each other. Since PC1 contains high positive loadings for GSSG, high scoring for PC1 indicate high concentrations for GSSG. Henceforward, the cluster on d2 was characterized by low GSSG concentrations in several tissues. Most interestingly, gastro-intestinal tissues such as the duodenum, distal jejunum and liver, were considered relatively more important in this PC. Nevertheless, positive loadings for GSH in liver, lung and kidney also indicate that the cluster of d2 is characterized by low levels of GSH in these organs. Next, PC2 discriminated subjects either belonging to d14 versus d0 and d2. Again, a significant DAY effect was found. In particular, d14 post-weaning was significantly different from d0 and d2 post-weaning (*p* < 0.001). No other factors or interactions significantly altered the scorings of PC2. This component had high positive loadings for the distal jejunal GSH concentration, and renal GSSG concentration. The cluster at d14 post-weaning was, thus, characterized by high GSH levels in the distal jejunal mucosa, and increased GSSG levels in the renal cortex.

Second, it is remarkable that variables with |r|> 0.5 within a principal component all have positive loadings, i.e., variables are not negatively related to each other. Bivariate analysis (results not shown) confirmed that within a tissue, GSH and GSSG levels are exclusively positively correlated (0.471 < r < 0.730; *p* ≤ 0.05). Likewise, GSH and GSSG concentrations each are exclusively positively correlated in between tissues (0.330 < r < 0.758; *p* ≤ 0.05). Additionally, the GSH/GSSG E_h_ in erythrocytes, liver, duodenal, and distal jejunal mucosa are positively correlated to each other (0.443 < r < 0.757; *p* < 0.01). However, the GSH redox status in lung tissue is negatively correlated to that in duodenal mucosa (r = 0.487; *p* < 0.01), distal jejunal mucosa (r = −0.516; *p* < 0.01), and liver (r = −0.496; *p* < 0.01). The renal cortical GSH redox status did not significantly correlate with the redox status in other tissues.

## 4. Discussion

### 4.1. The Weaning Transition Highly Affects the Glutathione Redox System

To our knowledge, this is the first study describing post-weaning GSH alterations in several tissues simultaneously. Other studies described similar events in either whole blood [26], plasma [27], erythrocytes [1,28], the small intestinal mucosa [1,28,29,30], and liver [28]. No information is available for lung or kidney tissue. From the current study, it is clear that weaning alters the GSH redox system. Weaning affected the GSH level in almost all measured tissues, and more specifically reduced GSH levels from d0 to d2 post-weaning with 22% to 80%. In addition, PCA analysis could clearly cluster piglets according to the day post-weaning, based on GSH and GSSG concentrations in the different tissues. One study reported similar events on d2 post-weaning, i.e., a decrease in erythrocyte GSH and unaffected duodenal GSH levels, where the latter was also found to increase on d12 post-weaning [1]. Nevertheless, the drop in distal jejunal GSH could not be confirmed by this earlier study. Wang et al. (2008) [29] and Li et al. (2014) [30] did report a drop in jejunal GSH at d7 post-weaning. This co-occurred with a more oxidized jejunal GSH redox status in both studies, which was not the case in the current study. Our earlier study did however describe a similar event in the duodenum at d5 post-weaning [1]. In lung tissue the outcome was quite different from other tissues. Pulmonary GSH levels remained low from d2 onwards, and GSSG concentrations dramatically increased from d2 to d14 post-weaning, resulting in a repeated increase of the GSH/GSSG E_h_ throughout the weaning transition. No other authors measured nor reported this event. Interestingly, Deneke et al. (1985) demonstrated that even a temporarily decrease of pulmonary GSH, attenuated by diethyl maleate, gave rise to morphological alterations affecting the functionality of the lung [31]. In addition, the GSH redox status exhibits regulatory function in the innate and adaptive immune response in the lung [32]. Finally also the GSH redox system in the renal cortex was affected by weaning, which could have critical importance in maintaining cell viability and functionality in the renal proximal tubule [33]. Although we did not further investigate whether the alterations afflicted functional or immunological adaptations in these tissues, these early biochemical events do potentially indicate relevant new targets for remediation in weaned piglets.

Apart from the fact that several authors report weaning induced alterations of the GSH redox system, it remains questionable if a clear and repeated response can be expected. Importantly, changes of the GSH redox system are considered to be spatiotemporal [3], and this is particularly entangled by the multifactorial character of the weaning transition. Inflammation and fasting [34,35], commonly observed in weaned piglets, could trigger oxidative stress. Still, the effects are expected to be highly variable between individual animals and even tissues. Additionally, interorgan translocation of the GSH pool even further complicates the interpretation [10,36]. Typically, this depends on the expression of the ectoenzyme γ-glutamyltransferase, which is most pronounced in the proximal tubular epithelium of the renal cortex and in the small intestinal epithelium [33]. These tissues, therefore, are the primary tissues extracting GSH on the luminal or brush-border plasma membrane. In our study, GSH levels were maintained in the duodenal mucosa at d2 post-weaning. Next, first-pass metabolism of cysteine [37] could also enabled this response in the duodenum. Finally biliary GSH efflux could have attributed to redox homeostasis in the duodenal mucosa [38]. Our earlier work demonstrated these principles in weaned piglets challenged with dietary lipid peroxides [28].

### 4.2. N-Acetyl-Cysteine Did Not Improve Tissue Glutathione Levels

It was hypothesized that NAC would be crucial for maintaining adequate GSH levels during the immediate post-weaning period, especially in fasted animals. Results of this study nevertheless indicate otherwise. Although NAC improved the duodenal GSH redox status, it did not increase GSH levels in any tissues, not even in the liver which is the main site of GSH synthesis [37]. Here, it is important to point out that fasting did not influence water intake throughout the study, hence, the NAC intake was similar for both fasted and non-fasted piglets. Unfortunately, this result cannot be confirmed by the literature, as studies reporting beneficial effects of NAC at 500 mg/kg diet in weaned piglets did not measure tissue GSH levels [15,16,17,18,19,20]. Yet, hepatic GSH levels were increased by NAC in pre-weaning intrauterine growth retarded piglets [39]. Song et al. (2016) also found an increase in intestinal GSH in LPS treated piglets at d20 post-weaning, supplemented L-cysteine at 250 and 500 mg/kg diet [40]. These doses are quite similar to the dose used in the current study, where NAC was applied at 200 mg/L water and the water-to-feed ratio was approximately 2.5 L/kg feed. Nevertheless, these studies included animals in an adequately fed status, which contrasts with the current study which focused on d2 post-weaning. Piglets in this phase typically undergo a period of malnutrition [23,24,41], and this aspect is imperative when investigating dietary responses in the weaned piglet. Therefore, to conclude from our results, NAC as a precursor for cysteine is potentially not the first or only limiting factor for GSH synthesis during the weaning transition. For example, GSH synthesis also requires glutamine, which could similarly be limited in supply [42]. Second, catalyzing the GSH synthesis also requires upregulation of the rate-limiting enzyme GCL [9]. Finally, ATP and NADPH availability could respectively restrict the synthetic capacity and the reduction of oxidized thiol-like GSSG and cystine, supporting a GSH increase [9].

### 4.3. Fasting Did Not Amplify Weaning Induced Alterations of the Glutathione Redox System

Although the immediate post-weaning growth check was significantly higher in fasted piglets, animal performance was not altered on d14 or d42 post-weaning. This evidence does not align with what was anticipated based on a vast amount of literature, i.e., fasting during the acute post-weaning period is detrimental for gut health, and thereby affects later performance during the weaning transition [23,24,41,43]. We did not observe a response of fasting on growth, feed intake or feed conversion in the period following fasting. This might lead to the conclusion that also unfasted piglets experienced a level of feed intake which was far too low to maintain normal homeostasis.

In the current study, fasting did not exhibit any significant effects on the GSH redox system. This does not align with research of Ushida et al. (2017) in rats, were jejunal GSH concentrations were significantly reduced upon 42 and 72 h fasting [7]. Additionally, Cho et al. (1981) indicated that fasting for 24 and 48 h reduced GSH levels of several tissues in rats, including the liver and the small intestine [44]. In the current study, we did however monitor decreased GSH levels in several tissues at d2 post-weaning, irrespective of fasting or *ad libitum* feeding. Bearing in mind the low feed intake in *ad libitum* fed animals, this observation might therefore be the result of an overall low feed intake in both fasted and *ad libitum* fed animals. In conclusion, it can be argued that fasting did not deteriorate animal performance, nor did it amplify the GSH redox imbalance normally caused by weaning.

## 5. Conclusions

In summary, the GSH redox status was affected in all tissues throughout the weaning transition. Accordingly, PCA analysis could clearly cluster animals by day post-weaning based on the tissue GSH and GSSG levels. Nevertheless, the nutritional status did not influence the GSH drop at d2 post-weaning, when comparing fasted with *ad libitum* fed piglets. Though, it must be pointed out that immediate post-weaning feed intake was also critically low in unfasted animals. In addition, NAC supplementation in the drinking water did not result in higher GSH levels in any tissue, not even in fasted animals, suggesting that cysteine availability was not the first or only rate limiting factor for GSH synthesis in the early post-weaning phase. Finally, NAC supplementation from d0–14 post-weaning did not improve animal performance until d42 post-weaning.

## Figures and Tables

**Table 1:** Chemical composition (calculated) and ingredients of the weaner and starter diet, fed to piglets from d0–d14 and d14–42 post-weaning, respectively.

Nutrients (g/kg as-Fed Basis)	Weaner Diet ^1^	Starter Diet ^2^
Dry matter	889.0	889.0
Crude protein	160.0	165.0
Ether extract	56.0	54.0
Crude fibre	35.0	40.0
Crude ash	54.5	57.0
Calcium	7.1	7.1
Phosphorus	5.5	5.5
Potassium	2.3	2.5
Lysine	11.9	11.7
Methionine	4.6	4.4

^1^ Major ingredients: Barley, sweet whey powder, wheat, toasted soybeans, extruded corn, wheat bran, sugar beet pulp, and wheat gluten meal (80% CP). The mineral and vitamin premix supplied as the following (per kg diet): vitamin A, 15,000 IU; vitamin D3, 2000 IU; vitamin E, 150 mg; vitamin C, 100 mg; choline chloride, 200 mg; Fe^2+^, 160 mg; Zn^2+^, 111 mg; Cu^2+^, 150 mg; Mn^2+^, 50 mg; Se^6+^, 0.42 mg; I^−^, 1.5 mg; 6-phytase, 750 FTU; endo-1,3(4)-beta-glucanase, 10 FBG; beta-xylanase, 190 FXU; *Bacillus licheniformes* 0.6 × 10^9^ CFU; *Bacillus subtilis*, 0.6 × 10^9^ CFU; clinoptilolite, 450 mg; bentonite montmorillonite, 250 mg; butylated hydroxytoluene, 52.95 mg; propyl gallate, 2.56 mg. ^2^ Major ingredients: Barley, wheat, corn, toasted soybeans, sweet whey powder, soybean meal (48% CP), wheat bran, sugar beet pulp, corn, wheat gluten meal (80% CP), fish oil, sodium chloride. The mineral and vitamin premix supplied as the following (per kg diet): vitamin A, 15 000 IU; vitamin D3, 2000 IU; vitamin E, 150 mg; vitamin C, 100 mg; choline chloride, 160 mg; Fe^2+^, 160 mg; Zn^2+^, 111 mg; Cu^2+^, 150 mg; Mn^2+^, 50 mg; Se^6+^, 0.42 mg; I^−^, 1.5 mg; endo-1,3(4)-beta-glucanase, 10 FBG; beta-xylanase, 190 FXU; *Bacillus licheniformes* 0.6 × 10^9^ CFU; *Bacillus subtilis*, 0.6 × 10^9^ CFU; clinoptilolite, 450 mg; bentonite montmorillonite, 350 mg; butylated hydroxyanisole, 2.41 mg; butylated hydroxytoluene, 52.95 mg; ethoxyquin, 1.56 mg; propyl gallate, 1.00 mg.

**Table 2:** Post-weaning animal performance of piglets either or not fasted from d0–2 (respectively FA+ and FA−) and either or not supplemented with *N*-acetyl cysteine (NAC) via the drinking water at 200 mg/L (respectively, NAC+ and NAC−) (*n* = 7).

	Treatment				SEM	*p*-Value		
	FA−NAC−	FA+NAC−	FA−NAC+	FA+NAC+		FA	NAC	FA × NAC
**Body weight (BW) [kg]**								
**d0**	7.05	7.05	7.05	7.06	0.01	0.880	0.846	0.746
**d2**	6.92	6.70	6.91	6.73	0.01	**<0.001**	0.768	0.342
**d14**	9.13	8.87	8.99	9.03	0.05	0.301	0.925	0.165
**d42**	23.04	22.14	22.64	22.71	0.21	0.335	0.845	0.259
**Average daily gain (ADG) [g/d]**								
**d0–2**	−64	−175	−72	−161	7	**<0.001**	0.849	0.465
**d2–14**	185	183	176	193	4	0.441	0.962	0.303
**d14–42**	498	478	490	491	6	0.457	0.823	0.410
**d0–42**	383	362	372	375	5	0.354	0.882	0.255
**Average daily feed intake (ADFI) [g/d]**								
**d0–2**	56	0	50	0	4	^1^	0.684	^1^
**d2–14**	252	239	243	256	4	0.961	0.619	0.151
**d14–42**	799	778	802	790	16	0.609	0.820	0.896
**d0–42**	589	568	588	582	10	0.474	0.744	0.700
**Average daily water intake (ADWI) [mL/d]**								
**d0–2**	329	322	311	308	17	0.899	0.646	0.954
**d2–14**	648	588	638	606	19	0.251	0.920	0.723
**d14–42**	2118	2451	2256	2314	115	0.405	0.997	0.557
**d0–42**	1525	1716	1617	1638	74	0.484	0.962	0.572
**Feed conversion ratio (FCR) [ADG/ADFI]**								
**d0–2**	−1.53		−1.18		0.17	^1^	0.612	^1^
**d2–14**	1.36	1.31	1.38	1.34	0.02	0.129	0.325	0.884
**d14–42**	1.61	1.62	1.65	1.61	0.03	0.881	0.791	0.680
**d0–42**	1.54	1.57	1.59	1.56	0.03	0.988	0.715	0.608
**Water to feed ratio (WFR) [ADWI/ADFI]**								
**d0–2**	9.03		7.49		1.62	^1^	0.643	^1^
**d2–14**	2.58	2.46	2.61	2.37	0.07	0.199	0.848	0.688
**d14–42**	2.69	3.20	2.86	2.93	0.17	0.393	0.888	0.525
**d0–42**	2.58	3.04	2.78	2.82	0.15	0.404	0.967	0.483

*p*-Values that reach significance (*p* ≤ 0.05) are indicated in bold. ^1^ Robust tests of equality of means cannot be performed because at least one group has zero variance.

**Table 3:** Significance levels for treatment effects on the glutathione redox system in piglets at d0, d2, and d14 post-weaning (DAY), either or not fasted from d0–2 (FA), and either or not supplemented with *N*-acetyl cysteine via the drinking water at 200 mg/L (NAC) (*n* = 4).

*p*-Value							
	DAY	FA	NAC	DAY × FA	DAY × NAC	FA × NAC	DAY × FA × NAC
**GSH**							
**Erythrocytes**	**0.002**	0.125	0.206	0.450	0.982	0.788	0.594
**Duodenum**	**<0.001**	0.952	0.480	0.552	0.973	0.905	0.725
**Distal jejunum**	**<0.001**	0.887	0.865	0.854	0.585	0.600	0.565
**Liver**	**<0.001**	0.740	0.877	0.698	0.745	0.634	0.505
**Lung**	**<0.001**	0.345	0.705	0.924	0.166	0.716	0.601
**Kidney**	**<0.001**	0.257	0.138	0.415	0.905	0.055	**0.038**
**GSSG**							
**Erythrocytes**	0.440	0.608	0.918	0.518	0.619	0.081	0.686
**Duodenum**	0.077	0.895	0.428	0.883	0.216	0.857	0.412
**Distal jejunum**	**0.027**	0.568	0.894	0.572	0.849	0.908	0.993
**Liver**	**<0.001**	0.320	0.844	0.736	0.501	0.167	0.418
**Lung**	**<0.001**	0.943	0.597	0.485	0.539	0.413	0.340
**Kidney**	**<0.001**	0.377	0.121	0.587	0.082	0.757	0.874
**GSH/GSSG E_h_**							
**Erythrocytes**	**<0.001**	0.222	0.060	0.866	0.713	**0.041**	0.276
**Duodenum**	**<0.001**	0.552	**0.044**	0.971	0.062	0.366	0.058
**Distal jejunum**	**<0.001**	0.944	0.985	0.623	0.763	0.531	0.731
**Liver**	**0.007**	0.709	0.945	0.673	0.537	0.775	0.076
**Lung**	**<0.001**	0.537	0.571	0.218	0.435	0.861	0.659
**Kidney**	**0.017**	0.821	0.725	0.766	0.175	0.454	0.149

*p*-Values that reach significance (*p* ≤ 0.05) are indicated in bold. GSH = glutathione; GSSG = glutathione disulphide; GSH/GSSG E_h_ = the glutathione redox status.
